# Breast Cancer (BC) and Severe COVID-19 (C-19) Outcomes: A Matched Analysis

**DOI:** 10.21203/rs.3.rs-3485880/v1

**Published:** 2023-12-14

**Authors:** Marija Sullivan, Xiudong Lei, Sharon H Giordano, Mariana Chavez-MacGregor

**Affiliations:** The University of Texas MD Anderson Cancer Center; The University of Texas MD Anderson Cancer Center; The University of Texas MD Anderson Cancer Center; The University of Texas MD Anderson Cancer Center

## Abstract

**Purpose:**

Patients with cancer receiving anticancer treatment have a higher risk of severe COVID-19 (C-19) outcomes. We examine the association between breast cancer (BC), recent treatment (systemic therapy, surgery, radiation), and C-19 outcomes.

**Methods:**

Retrospective matched cohort study using the Optum^®^ de-identified COVID-19 Electronic Health Record dataset (2007–2022). Patients with C-19 were categorized into: No cancer, BC with recent treatment, and BC without recent treatment and matched based on age, C-19 diagnosis date, and comorbidity score. We evaluated 30-day mortality, mechanical ventilation, intensive care unit (ICU) stay, and hospitalization. A composite outcome including all outcomes was analyzed. Multivariable logistic regression models were used.

**Results:**

2200 matched triplets (1:1:10) of patients with BC recently treated, BC not recently treated, and no cancer were included. Rates of adverse outcomes improved in 2021 compared to 2020. Compared to patients without cancer, those with BC recently treated had a similar risk of adverse outcomes, while patients with BC not recently treated had a lower risk of ICU stay and hospitalization. Using the composite variable, BC recently treated had similar outcomes (OR = 1.02; 95%CI 0.93–1.11) to patients without cancer, while BC patients not recently treated had better outcomes (OR = 0.66; 95%CI 0.59–0.74). Among patients with BC, chemotherapy within 3-months was associated with a higher risk of hospitalization (OR = 2.30; 95%CI 1.76–2.99) and composite outcome (OR = 2.11; 95%CI 1.64–2.72).

**Conclusion:**

Patients with BC have a similar risk of adverse C-19 outcomes compared to patients without cancer. Among patients with BC, recent chemotherapy was associated with a higher risk of hospitalization.

## Background

The COVID-19 (C-19) pandemic has had a profound and evolving impact on healthcare systems worldwide. As of June 2023, more than 760 million confirmed cases of C-19 and 6.9 million deaths have been reported globally [1]. Older age and comorbid conditions, particularly cardiovascular disease, diabetes, hypertension, and obesity, have been implicated in an increased risk of severe infection and worse C-19 outcomes [2–7]. Patients with cancer often have compromised immune systems, related to their cancer or cancer treatment, which increases their risk for infection. They also often have multiple comorbid conditions; thus, it is not surprising that several studies have found that patients with cancer have a higher risk of severe C-19 outcomes [8–12]. The risk of severe outcomes may vary with cancer type, with studies suggesting that those with hematologic malignancies have a higher risk of poor outcomes related to C-19 infection [10,13,14]. Vaccination has been effective in preventing infection and progression to severe disease, although efficacy studies are still ongoing for the various vaccines; in particular, examining how they will fare against recent C-19 variants [15]. It has previously been shown that C-19 outcomes in patients with breast cancer (BC) may depend more on presence of medical comorbidities, such as obesity, hypertension, and diabetes, than on the diagnosis and management of breast cancer itself [16]. In this study, we examine the association between BC, recent treatment modality, and adverse C-19 outcomes.

## Methods

### Data Source

In this retrospective matched cohort study, we queried the Optum^®^ de-identified COVID-19 Electronic Health Record dataset (2007–2022) to include patients diagnosed with C-19 between 01/01/2020 and 12/20/2021. The data were obtained from Optum^®^ COVID-19 data, which captures point-of-care diagnostic data that are specific to C-19, such as patient-level and clinical results from both inpatient and ambulatory settings. Race and ethnicity data were self-reported in the Optum^®^ COVID-19 data.

This study was granted approval by the MD Anderson Cancer Center Institutional Review Board, which considered the study exempt from obtaining patient informed consent based on its code of regulations. We followed the Strengthening the Reporting of Observational Studies in Epidemiology (STROBE) reporting guideline.

### Cohort Selection

We identified patients with C-19 using diagnosis codes, Healthcare Common Procedure Coding System (HCPCS) codes, Logical Observation Identifiers Names and Codes (LOINC), and laboratory test names as previously Described **(Supplementary Table-1)** [4]. We defined a positive test result for C-19 via polymerase chain reaction, antigen test, or serologic confirmation. We used the earliest collection, test, or result date as the C-19 diagnosis date between 1/1/2020 and 12/20/2021. The last follow-up date was January 20, 2022.

The eligible population included all female patients ≥ 18 years diagnosed with C-19 (n = 561,549). For patients with BC, we used the International Classification of Diseases 9th and 10th Revision (ICD-9 and ICD-10) diagnosis codes to identify those who had 2 or more BC diagnosis codes that were at least 30 days apart within 1 year before C-19 diagnosis date (n = 5,571). For controls without cancer, we included those with no cancer diagnosis code within 1 year prior to C-19 diagnosis (n = 528,626). We further categorized patients with BC according to recent (within 3 months prior to C-19 diagnosis) anticancer treatment (surgery, radiation therapy, chemotherapy, immunotherapy, or endocrine therapy), as not recently treated and recently treated.

We matched (1:1:10) patients with BC and controls without cancer based on age, comorbidity score, and date of C-19 diagnosis: **Group 1** included patients with BC and recent cancer treatment (referred to as BC recently treated); **Group 2** included patients with BC and no recent treatment (BC not recently treated); **Group 3** included patients without cancer (non-cancer controls) (**eFigure-1**).

### Outcomes and Covariates

Outcomes of interest included mortality, mechanical ventilation, intensive care unit (ICU) stay, and hospital admission within 30 days of C-19 diagnosis. Death data were obtained from the Social Security Administration Death Master File, including month and year of death; therefore, date of death was set as the 15th of the month. To better capture mortality, we extended follow-up to the next month among patients who were diagnosed with C-19 after the 15th of the month. We identified mechanical ventilation using HCPCS codes or ICD-10 procedure codes. ICU stay was defined as hospitalization in an ICU without mechanical ventilation.

Demographic and patient characteristics included age (18–49, 50–59, 60–69, 70–79, ≥ 80), race and ethnicity (Hispanic, non-Hispanic Black, non-Hispanic White, Others including non-Hispanic Asian and unknown), insurance type (commercial, Medicare, Medicaid, other or unknown) and region (Midwest, Northeast, South, West, unknown). Clinical characteristics included COVID-19 diagnosis period, comorbidities excluding cancer [17,18], severe obesity (body mass index ≥ 40 kg/m^2^), skilled nurse facility (SNF) stay within 3 months before C-19 diagnosis, and C-19 vaccine before diagnosis. Among patients with BC, cancer-related therapy was identified using HCPCS codes **(Supplementary Table-1)** based on the National Cancer Institute Targeted Cancer Therapies Fact Sheet [19]. We defined metastatic disease using both diagnosis codes and drugs that treated metastatic disease in the 3 months before C-19 diagnosis.

We analyzed the risk of 30-day outcomes using multivariable logistic regression models. Variables in the model were selected based on a backward selection method and clinical relevance and included C-19 diagnosis period, age, race and ethnicity, severe obesity, Charlson-Deyo comorbidity index score, recent skilled nursing facility stay, insurance type, and region. A composite ordinal outcome including all outcomes was evaluated (higher score means more severe outcome: hospitalization = 1, ICU = 2, mechanical ventilation = 3, death = 4). Results are presented as odds ratios (OR) and 95% confidence intervals (CI).

Analyses were conducted using SAS, version 9.4 (SAS Institute), and R, version 4.0.5 (R Foundation for Statistical Computing). All tests were 2 sided, with a statistical significance level of P = .05.

## Results

**Figure 1:**
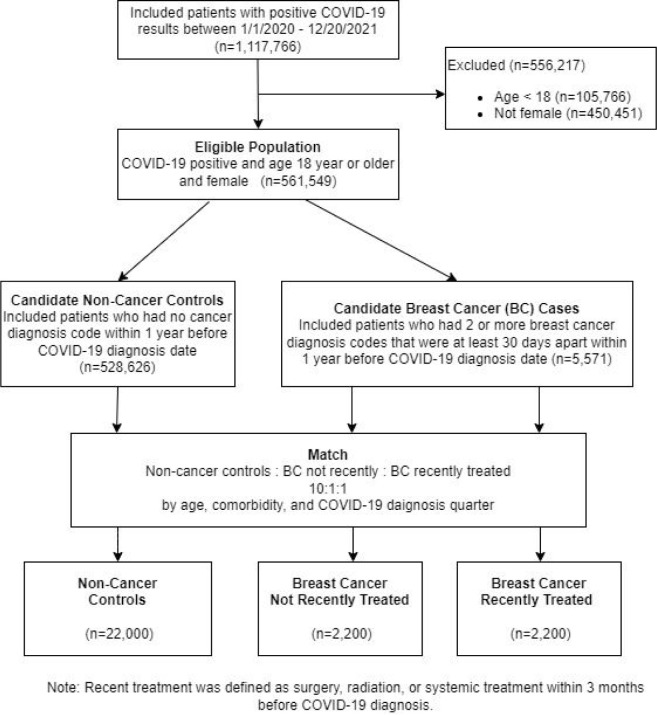
Flow Diagram of Cohort Selection Using the Optum^®^ COVID-19 data*

We matched 2200 triplets (1:1:10) of BC treated, BC not treated (Figure-1), and non-cancer patients (Table-1). Median age was 65 years (IQR 55–74 years). In total, 3.6%, 5.8%, 5.5% non-cancer, BC treated, BC not treated received vaccine before their C-19 diagnosis. Among the 1033 (3.8% out of 26,400) patients who had vaccine before C-19 diagnosis, 195 patients had 1 dose of C-19 vaccine including 36 [18.6%] Janssen vaccine, 70 (36%) Moderna vaccine, 82 (42%) Pfizer vaccine, and 7 (3.6%) other vaccine. The remaining 838 patients had 2 or more doses of vaccine including 352 (42%) Moderna, 456 (54%) Pfizer, 1 patient with 2 doses of Janssen, and the rest 29 patients with mixed vaccines.

**Figure 2:**
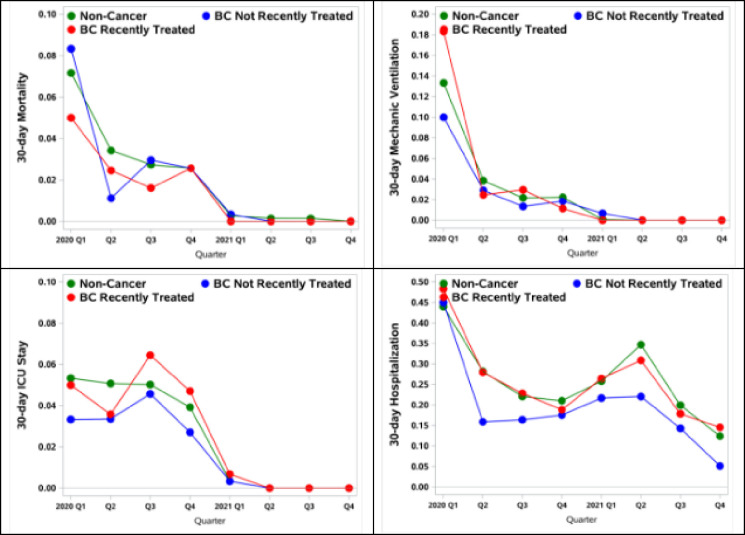
Unadjusted Quarterly Rates of 30-day Mortality, Mechanical Ventilation, ICU Stay, and Hospitalization Among Non-Cancer Patients, Patients with Breast Cancer Not Recently Treated and Patients With Breast Cancer Recently Treated

Except for hospitalization, rates of adverse outcomes in 2021 dropped precipitately from 2020 (yearly average mortality: 2.9–0.2%; mechanical ventilation: 3–0.1%; ICU stay: 4.5–0.2%; hospitalization: 23.6–22.4%) (Figure-2). There was a sharp drop from Quarter (Q) 1 to Q2 in 2020 because of improved C-19 preparedness, and from 2020 to 2021 after a vaccine first became available and management of patients with C-19 improved. When stratifying by cohorts, for the non-cancer cohort, 30-day mortality was 7.2%, 3.4%, 2.7%, and 2.6% in 2020 Q1-Q4, and the rate dropped to 0.3% in Q1 2021. For patients with BC not recently treated, the 30-day mortality was 8.3 %, 1.1%, 3.0%, 2.6%, in 2020 Q1-Q4 and 0.3% in 2021 Q1. For patients with BC recently treated, the 30-day mortality was 5.0%, 2.4%, 1.6%, 2.6%, in 2020 Q1-Q4 and 0% 2021 Q1.

Compared to patients without cancer, those with BC recently treated had similar risk of adverse outcomes (OR = 0.80–1.07, p ≥ 0.21), while patients with BC not recently treated had lower risk of ICU stay (OR = 0.71; 95%CI 0.53–0.94, p = 0.02) and hospitalization (OR = 0.64; 95%CI 0.57–0.73, p < 0.001) and similar risk of mortality (OR = 0.83; 95%CI 0.60–1.16, p = 0.28) and mechanical ventilation (OR = 0.81; 95% CI0.58–1.13, p = 0.22) (Table-2). Using the composite ordinal variable, patients with BC recently treated had similar outcomes (OR = 1.02; 95%CI 0.93–1.11, p = 0.67) to those without cancer, and patients with BC not recently treated had better outcomes (OR = 0.66; 95%CI 0.59–0.74, p < 0.001), an observation that was mostly driven by lower rates of hospitalization and ICU stay.

Sensitivity analysis regrouping patients with endocrine therapy only (N = 1225) into the group of patients categorized as not having received treatment, showed similar results (**Supplemental Table-2**). Subgroup analysis among patients with BC included 2,200 pairs of patients with and without recent (within 3 months) treatment. Among patients recently treated, 491 (22%) patients received recent chemotherapy, 109 (5%) received recent immunotherapy, 193 (9%) received both chemotherapy and immunotherapy, 1347 (61%) received endocrine therapy, 181 (4.1%) underwent surgery, and 189 (4.3%) received radiation. In the multivariable analysis, compared to patients with no systemic therapy (Table-3), receipt of chemotherapy within 3 months of C-19 diagnosis was associated with a higher risk of hospitalization (OR = 2.30; 95%CI 1.76–2.99, p < 0.001), ICU stay (OR = 1.81; 95%CI 1.02–3.23, p = 0.04), and consequently a higher risk of composite ordinal outcome (OR = 2.11; 95%CI 1.64–2.72, p < 0.001). Receipt of immunotherapy was associated with a higher risk of ICU stay (OR = 2.60; 95%CI 1.10–6.17, p = 0.03) but other outcomes including composite ordinal outcome (OR = 1.30; 95%CI 0.80– 2.09, p = 0.29) were comparable to patients not recently treated with systemic therapy. When combined, receipt of both chemotherapy and immunotherapy was associated with a higher risk of hospitalization (OR = 1.51; 95%CI 1.04–2.19, p = 0.03), ICU stay (OR = 3.21; 95 CI 1.68–6.13, p < 0.001), and composite ordinal outcome (OR = 1.67; 95%CI 1.18–2.37, p = 0.004). Patients who recently received endocrine therapy were associated with a higher risk of hospitalization (OR = 1.39; 95%CI 1.16–1.66, p < 0.001) and subsequently a higher risk of composite ordinal outcome (OR = 1.28; 95%CI 1.07–1.52, p = 0.006) that was driven by hospitalization. We conducted sensitivity analysis by grouping patients who were treated with endocrine therapy only into patients with BC with no recent treatment. Results for 30-day and composite outcomes according to recent systemic treatment were shown in **Supplementary Table-3**. Models were additionally adjusted for metastatic disease. Results were similar, however attenuated. Patients who underwent recent surgery or radiation (within 3 months of C-19 diagnosis) did not have increased risk of adverse C-19 outcomes, surgery was associated with decreased risks of hospitalization (OR = 0.55; 95%CI 0.35–0.86, p = 0.009), and subsequently with decreased risks of the ordinal outcome (OR = 0.59; 95%CI0.39–0.91, p = 0.02).

## Discussion

In this large cohort of patients with C-19 categorized as BC treated, BC not treated, and without cancer, we observed that patients with BC had similar outcomes to the matched non-cancer cohort. Over the last few decades, patients with BC have had improved prognosis and survival due to widespread mammography screening and improved treatment [20]. This improvement in outcomes explains the similarities found between patients with BC and controls without cancer. Interestingly, among patients with BC not recently treated, outcomes including ICU stay and hospitalization were better compared to non-cancer controls. This may be related to the good general health of BC survivors or to increased health vigilance of patients with history of cancer without the consequence of potential immunosuppression related to ongoing treatment or active disease. Patients with history of BC have been shown to have similar general health and quality of life compared to controls without cancer [21]. This may be related to the “teachable moment” that a diagnosis of cancer may serve with an opportunity to address preventative health strategies for chronic health conditions following acute management of their cancer [22]. Among patients with BC, recent treatment with both chemotherapy and immunotherapy (within 3 months of C-19 diagnosis) was associated with a higher rate of both ICU stay and hospitalization compared to those not recently treated. Data evaluating cancer-directed therapy and its association with adverse C-19 outcomes has been mixed with some studies suggesting similar outcomes between patients with cancer and those without cancer [23–25] and others suggesting worse outcomes for patients with cancer [8,12,26,27]. The mechanism behind increased susceptibility to worse outcomes in patients receiving chemotherapy or immunotherapy can be postulated – that of a compromised or altered immune system – but it may be that the heterogeneity of cancer types and cancer-directed therapies is leading to mixed data. In our study, patients with breast cancer who had recent chemotherapy or immunotherapy had worse outcomes than patients with breast cancer not recently treated. Interestingly, patients that underwent recent surgery or radiation (within 3 months of C-19 diagnosis) did not have increased risk for adverse C-19 outcomes. Though we had small numbers of patients in the cohort that underwent recent surgery or radiation (4.1% underwent surgery and 4.3% received radiation), these findings are consistent with prior studies indicating no increased risk of mortality from C-19 after recent cancer surgery or radiation [28,29].

Given that endocrine therapy has a different side effect profile when compared to immunotherapy and chemotherapy and is most often used in the adjuvant setting to reduce the risk of recurrence following local therapy, we re-grouped patients who were treated with endocrine therapy only into BC patients with no recent treatment. To exclude confounding by patients receiving endocrine therapy for metastatic disease, we adjusted for metastatic disease since we understand that advanced disease could be a factor associated with severity of C-19 as previously reported [4]. In this sensitivity analysis, we observed similar results, though attenuated, suggesting that the link between advanced disease and adverse C-19 outcomes in patients with BC may be related to immunosuppressive status or poor performance status. One recent study found no increased risk for C-19 infection in patients with BC treated with chemotherapy, but it did find an association between metastatic disease and mortality following C-19 infection [23].

As expected, we found that adverse outcomes, specifically ICU stay, mechanical ventilation and mortality, improved in 2021 when compared to 2020. This positive change stems from increased understanding of C-19 disease and increased availability of treatment and resources. In our cohort, the number of patients with BC who were vaccinated prior to C-19 diagnosis was low, making it difficult to interpret any association between vaccine status and C-19 outcomes.

### Limitations

Despite the strength of a large cohort size, this EHR-based study is limited by its reliance on ICD-9 and ICD-10 codes to identify patients with breast cancer, which may lead to false positives. Specifically, human error in coding may lead to over or under-documentation of cancer and cancer-related treatment. While a strength of the Optum^®^ COVID-19 data is its geographic diversity of patients in the US, it is limited to capturing patients with insurance and thus the data is less generalizable when considering patients that are uninsured. In addition, while the dates included in this study are limited, they represent the peak of the C-19 pandemic and were selected as they represent a time before the widespread use of the vaccines was incorporated and newer and more effective therapies were incorporated. We also acknowledge that our results are originating from observations in the US population and thus, our results need to be carefully interpreted from a global perspective.

## Conclusion

Patients with BC have a similar risk of adverse C-19 outcomes compared to noncancer patients. Subgroup analysis among patients with BC suggests that recent chemotherapy was associated with a higher risk of hospitalization among patients with BC.

## Figures and Tables

**Table-1.** Characteristics of matched patients diagnosed with breast cancer and COVID-19 identified in the Optum^®^ COVID-19 data (N=26,400)

	Non-Breast Cancer (N=22,000) N (Column %)	Breast Cancer Not Recently Treated (N=2,200) N (Column %)	Breast Cancer Recently Treated (N=2,200) N (Column %)	P value
**Diagnosis period**				
2020 January-March	600 (2.7)	60 (2.7)	60 (2.7)	1
2020 April-June	4470 (20.3)	447 (20.3)	447 (20.3)	
2020 July-September	3720 (16.9)	372 (16.9)	372 (16.9)	
2020 October-December	7010 (31.9)	701 (31.9)	701 (31.9)	
2021 January-March	2950 (13.4)	295 (13.4)	295 (13.4)	
2021 April-June	680 (3.1)	68 (3.1)	68 (3.1)	
2021 July-September	1400 (6.4)	140 (6.4)	140 (6.4)	
2021 October-December	1170 (5.3)	117 (5.3)	117 (5.3)	
**Age, years**				
Median (IQR)	65 (55–74)	65 (55–74)	65 (55–74)	1
18–49	3100 (14.1)	310 (14.1)	310 (14.1)	
50–59	4940 (22.5)	494 (22.5)	494 (22.5)	
60–69	5850 (26.6)	585 (26.6)	585 (26.6)	
70–79	5360 (24.4)	536 (24.4)	536 (24.4)	
≥80	2750 (12.5)	275 (12.5)	275 (12.5)	
**Charlson-Deyo Comorbidity Index score**				
0	9550 (43.4)	955 (43.4)	955 (43.4)	1
1	4400 (20)	440 (20)	440 (20)	
≥ 2	8050 (36.6)	805 (36.6)	805 (36.6)	
**Race and ethnicity**				
Hispanic	1904 (8.7)	145 (6.6)	134 (6.1)	<0.001
Non-Hispanic Black	3138 (14.3)	334 (15.2)	348 (15.8)	
Non-Hispanic White	14524 (66)	1554 (70.6)	1561 (71)	
Other or unknown	2434 (11.1)	167 (7.6)	157 (7.1)	
**Severe obesity (BMI ≥ 40 kg/m^2^)**				
No	19185 (87.2)	1985 (90.2)	1972 (89.6)	<0.001
Yes	2815 (12.8)	215 (9.8)	228 (10.4)	
**SNF stay within 3 months before COVID-19 diagnosis**				
No	21615 (98.3)	2139 (97.2)	2145 (97.5)	<0.001
Yes	385 (1.8)	61 (2.8)	55 (2.5)	
**COVID19 vaccine before diagnosis**				
No	21217 (96.4)	2072 (94.2)	2078 (94.5)	<0.001
Yes	783 (3.6)	128 (5.8)	122 (5.5)	
**Insurance type**				
Commercial	12131 (55.1)	1249 (56.8)	1288 (58.5)	<0.001
Medicare	6312 (28.7)	688 (31.3)	675 (30.7)	
Medicaid	1214 (5.5)	134 (6.1)	130 (5.9)	
Other or unknown	2343 (10.7)	129 (5.9)	107 (4.9)	
**Region**				
Midwest	10084 (45.8)	1184 (53.8)	1199 (54.5)	<0.001
Northeast	4684 (21.3)	463 (21)	508 (23.1)	
South	4536 (20.6)	332 (15.1)	270 (12.3)	
West	1039 (4.7)	108 (4.9)	101 (4.6)	
Unknown	1657 (7.5)	113 (5.1)	122 (5.5)	

* Abbreviations: BMI=Body Mass Index; SNF=Skilled Nursing Facility

**Table-2.** Adjusted matched group 30-day and composite outcomes according to cancer status and recent breast cancer treatment^a^ (3 months before COVID-19 diagnosis).

Matched Cohort	Mortality		Mechanical ventilation		ICU stay		Hospitalization		Composite Ordinal Outcome	
	OR (95% CI)	P	OR (95% CI)	P	OR (95% CI)	P	OR (95% CI)	P	OR (95% CI)	P
**No cancer**	1		1		1		1		1
**BC no treatment**	0.83 (0.6-1.16)	0.28	0.81 (0.58-1.13)	0.22	0.71 (0.53-0.94)	0.02	0.64 (0.57-0.73)	<0.001	0.66 (0.59-0.74)	<0.001
**BC treatment** ^b^	0.8 (0.57-1.13)	0.21	0.88 (0.63-1.22)	0.43	1.07 (0.84-1.37)	0.57	0.98 (0.88-1.1)	0.74	1.02 (0.93-1.11)	0.67

BC: Breast cancer; OR: odds ratio; ICU: intensive care unit.

a Variables in model included: C-19 diagnosis period, age, race and ethnicity, severe obesity (BMI≥40), Charlson- Deyo comorbidity index score, recent skilled nursing facility stay, insurance type, and region.

b Breast cancer treatment included surgery, radiation, chemotherapy, immunotherapy, and endocrine therapy.

**Table-3.** Subgroup analysis of 30-day and composite outcomes among patients with breast cancer according to recent systemic treatment^a^ (3 months before COVID-19 diagnosis).

Systemic Therapy	Mortality		Mechanical Ventilation		ICU Stay		Hospitalization		Composite Ordinal Outcome	
	OR (95% CI)	P	OR (95% CI)	P	OR (95% CI)	P	OR (95% CI)	P	OR (95% CI)	P
**No recent systemic therapy**	1		1		1		1		1	
**Chemotherapy**	1.35 (0.65-2.82)	0.42	1.86 (0.88-3.93)	0.11	1.81 (1.02-3.23)	0.04	2.3 (1.76-2.99)	<0.001	2.11 (1.64-2.72)	<0.001
**Immunotherapy**	1.49 (0.43-5.16)	0.53	0.63 (0.08-4.87)	0.65	2.6 (1.1-6.17)	0.03	1.26 (0.77-2.08)	0.36	1.3 (0.8-2.09)	0.29
**Chemo-immunotherapy**	1.95 (0.85-4.5)	0.12	1.06 (0.34-3.31)	0.92	3.21 (1.68-6.13)	<0.001	1.51 (1.04-2.19)	0.03	1.67 (1.18-2.37)	0.004
**Endocrine therapy**	0.52 (0.27-0.98)	0.043	1.17 (0.68-1.99)	0.57	1.05 (0.67-1.64)	0.84	1.39 (1.16-1.66)	<0.001	1.28 (1.07-1.52)	0.006
**Surgery (yes vs no)**	1.03 (0.32-3.31)	0.96	NA^b^	0.97	0.92 (0.36-2.32)	0.85	0.55 (0.35-0.86)	0.009	0.59 (0.39-0.91)	0.02
**Radiation (yes vs no)**	1.93 (0.73-5.1)	0.18	0.56 (0.11-2.86)	0.5	1.29 (0.58-2.86)	0.53	1.13 (0.77-1.64)	0.53	1.18 (0.82-1.69)	0.37

Abbreviations: BC = breast cancer; OR = odds ratio; CI = confidence interval; ICU = intensive care unit.

a Variables in model included: Diagnosis period, age, race and ethnicity, severe obesity (BMI≥40), Charles-Deyo comorbidity index score, recent skilled scaled nursing facility stay, insurance type, and region.

b No valid estimates because of no event for surgery group.

## Data Availability

The source data for this study was Optum^®^ COVID-19 data, and hence we are not allowed to share the licensed data publicly. However, the same data used in this study are available for purchase by contracting with Optum (contact at: http://www.optum.com/solutions/data-analytics/data/real-world-dataanalytics-a-cpl/claims-data.html).
